# Impact of the Sulfurized Polyacrylonitrile Cathode Microstructure on the Electrochemical Performance of Lithium–Sulfur Batteries

**DOI:** 10.1002/advs.202415436

**Published:** 2025-02-22

**Authors:** Robin Moschner, Martina Gerle, Timo Danner, Esther Kezia Simanjuntak, Peter Michalowski, Arnulf Latz, Maryam Nojabaee, Arno Kwade, K. A. Friedrich

**Affiliations:** ^1^ Institute for Particle Technology Technische Universität Braunschweig Volkmaroder Straße 5 D‐38104 Braunschweig Germany; ^2^ Institute of Engineering Thermodynamics German Aerospace Center (DLR) Pfaffenwaldring 38‐40 D‐70569 Stuttgart Germany; ^3^ Helmholtz Institute Ulm (HIU) Electrochemical Energy Storage D‐89081 Ulm Germany; ^4^ Institute of Electrochemistry Ulm University D‐89081 Ulm Germany; ^5^ Institute for Building Energetics Thermotechnology and Energy Storage (IGTE) University of Stuttgart Pfaffenwaldring 31 D‐70569 Stuttgart Germany

**Keywords:** calendering, electrochemical characterization, electrochemical impedance spectroscopy, Lithium–sulfur battery, mechanical characterization, numerical simulation, sulfurized polyacrylonitrile

## Abstract

The growing demand for advanced energy storage systems requires the development of next‐generation battery technologies with superior energy density and cycle stability, with lithium–sulfur (Li–S) batteries representing a promising solution. Sulfur‐containing polyacrylonitrile cathodes (SPAN) for Li–S batteries are a significant advancement for this next‐generation battery chemistry, addressing the major issue of limited cycle life encountered in conventional carbon/sulfur composite cathodes. In the presented study, the influence of available ionic and electronic conduction pathways within the cathode on the electrochemical performance of SPAN‐based Li–S batteries is studied in details. To this end, a series of SPAN cathodes with different microstructures is prepared by adapting the compression degree of calendering. Mechanical and morphological characterizations confirm a pronounced springback effect due to a characteristic elastic deformation behavior of SPAN. Electrochemical impedance spectroscopy (EIS) shows increased cathode impedance values with multiple overlapping processes in the high‐ to mid‐frequency region in highly compressed SPAN cathodes. Moreover, while the (first) discharge capacity is unaffected, the subsequent charge capacity decreases substantially for highly compressed cathodes. The electrochemical experiments and electrochemical continuum simulations confirm that this phenomenon is mainly due to the disturbance of the electronic percolation pathways caused by the springback behavior during calendering.

## Graphical abstract

SPAN is a promising alternative sulfur cathode material for next‐generation batteries. However, the impact of the cathode microstructure on the electrochemical performance is not fully understood yet. Herein, the cathode microstructure is varied applying calendering. The influence of cathode porosity and carbon additive distribution enabling ionic and electronic conduction on the accessible capacity and rate capability is studied comprehensively. The observed structural and electrochemical changes are supported by simulative studies.

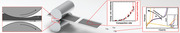

## 1 Introduction

The fast‐growing electrification of the mobility sector, the rising demand of stationary storages as well as the need for more powerful consumables ask for more sustainable, high energy, and durable electrochemical energy storage systems. Classical Lithium‐Ion‐Batteries (LIBs) have been significantly improved in energy and power density, longevity, and cost since their market introduction in the 1990s. However, their potential is limited by intrinsic specific energy constraints, rendering the absolute essence for the development of battery technologies based on conversion cathode materials, which theoretically are offering much higher specific capacities.^[^ ^1^, ^2^, ^3^, ^4^, ^5^ ^]^ Li‐S batteries are among the promising candidates within next‐generation batteries, offering a theoretical specific energy of 2600 Wh kg^−1^, and projected practical one of 600 Wh kg^−1^, significantly surpassing state‐of‐the‐art LIB capabilities. Moreover, sulfur is cost effective, naturally abundant, and non‐toxic.

However, practical application of Li‐S batteries is hindered by challenges including low active material conductivity, significant volume changes during cycling, reduced active material utilization, and poor cycle stability.^[^ ^6^, ^7^ ^]^ To address these issues, different approaches have been investigated elaborately, including the physical or chemical entrapment of sulfur in a meso‐ and microporous carbon host or in a shell‐structure,^[^ ^8^, ^9^, ^10^, ^11^, ^12^, ^13^ ^]^ modified electrolytes,^[^ ^14^, ^15^, ^16^, ^17^, ^18^ ^]^ redox mediators^[^ ^10^, ^19^, ^20^, ^21^ ^]^ or the generation of protective interphases on both, cathode^[^ ^22^, ^23^, ^24^ ^]^ or anode side.^[^ ^25^, ^26^ ^]^ Among these methods, one that has gained increased attention recently is to covalently bind the active material to a polymeric backbone, such as polyacrylonitrile (PAN).^[^ ^27^, ^28^, ^29^ ^]^ In SPAN cathodes, sulfur is mainly bound in form of bridging chains (‐S_x_‐, 3 ≤ x ≤ 5) to the vulcanized PAN‐backbone, achieving 30 to 45 wt.% sulfur loading.^[^ ^30^, ^31^, ^32^, ^33^ ^]^ Due to the stable structure, a direct solid‐solid conversion is enabled, hindering the sulfur dissolution and consequential polysulfide shuttle. Moreover, the material allows buffering of volume expansion, offers good thermal stability, flame retardancy and shows compatibility to various electrolytes and anodes.^[^ ^30^, ^34^, ^35^, ^36^ ^]^ Outstanding cycling stability as well as high C‐rate performance for SPAN cathodes in various ether and carbonate electrolytes without any polysulfide formation are reported.^[^ ^31^, ^37^, ^38^ ^]^

Extensive research was carried out regarding redox pathways of SPAN cathodes,^[^ ^28^, ^39^, ^40^, ^41^, ^42^ ^]^ synthesis,^[^ ^43^, ^44^, ^45^, ^46^ ^]^ electrode composition as well as improvement of cycling stability^[^ ^47^, ^48^, ^49^, ^50^, ^51^, ^52^, ^53^ ^]^ and sulfur amount.^[^ ^31^, ^38^, ^54^ ^]^ Specifically, it has been shown how the processing and synthesis of the SPAN active material affect the electrochemical performance, i.e., accessible capacity.^[^ ^37^, ^43^, ^55^, ^56^, ^57^ ^]^ Additionally, the impact of different conductive^[^ ^48^, ^50^, ^58^, ^59^ ^]^ and catalytic^[^ ^52^, ^59^, ^60^, ^61^, ^62^, ^63^, ^64^, ^65^ ^]^ additives and binders^[^ ^47^, ^49^, ^66^, ^67^ ^]^ has been investigated by various research groups.

However, only a few studies have examined the impact of SPAN‐based cathode processing characteristics such as grinding/milling, slurry preparation,^[^ ^56^, ^68^ ^]^ electrode drying or calendering.^[^ ^69^ ^]^ These factors affect the microstructure of the cathode, including particle size distribution, porosity, and the distribution of conductive additives. The aforementioned features of the cathode microstructure determine the degree of sulfur utilization, ionic and electronic transport pathways, and the ability to accommodate volume changes, all of which are crucial for the accessible capacity, rate capability, and cyclability of the cell.^[^ ^30^, ^48^, ^57^ ^]^ For instance, adequate void space within the cathode structure can reduce tortuosity and can help to accommodate the expansion and shrinkage of the active material during cycling, mitigating the mechanical stress and maintaining structural integrity and performance stability.^[^ ^51^ ^]^ On the other hand, reducing porosity increases the electrode density, electronic conductivity, bonding strength with the current collector, and the energy density of the cell. Therefore, the electrodes pore structure is usually optimized using calendering, a standard and cost‐effective process in conventional LIB production.^[^ ^70^, ^71^, ^72^, ^73^ ^]^

Building up on a previous study, where we investigated the influence of the slurry mixing step on the electrode properties of SPAN,^[^ ^68^ ^]^ this work provides insights into the calendering of SPAN cathodes. To this end, we investigate the impact of different calendering degrees on the electrode morphology and achievable capacity of SPAN‐based Li–S cell. Combining in‐depth mechanical and morphological characterization, elaborate electrochemical studies and detailed numerical simulations, we enable deep insight into influential structure‐related characteristics of the active material on the electrochemical performance. In this respect, the impact of the calendering process on the adhesion strength, electronic conductivity, porosity and plasticity of the electrode and their correlation to the electrode impedance response as well as cycling overpotentials, maximum capacity, and cyclability are discussed in details.

## 2 Results

Calendering is employed for classical LIB electrodes to enhance homogeneity and elasticity and to reduce the pore volume, thereby increasing energy density and improving electronic conductivity, especially for low‐conductive cathodes. During the calendering process, the applied line loads lead to particle rearrangements and structural compression. This in turn leads to changes of the adhesion strength, electronic conductivity, and plasticity of the electrode as well as the pore size distribution. For classical LIB electrodes like graphite anodes and NCM cathodes, a moderate calendering leads to the decrease of adhesion and an increase in the resistivity of the electrode due to the particle rearrangement and the disruption of the established networks within the electrodes. However, with higher grades of calendering, both adhesion and electronic conductivity improve again as the increased compression of the electrode structure enhances interparticle contacts, thereby improving the networking and percolation within the electrode.^[^ ^70^, ^71^ ^]^ Simultaneously, pore size and pore volume, particularly the interparticle porosity of the active material, are reduced.^[^ ^52^ ^]^ Additionally, the mechanical deformation behavior becomes increasingly elastic as the plastic deformation capability is exhausted during compression.^[^ ^74^ ^]^

**Figure 1:**
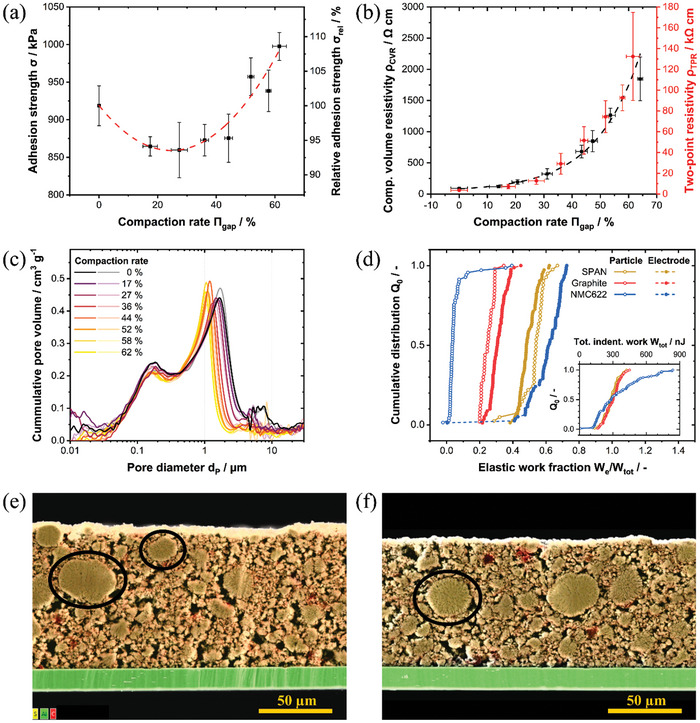
a) Adhesion strength of SPAN as function of compression grade; b) Multi‐point composite (black) and two‐point (red) electrical resistivity of SPAN electrodes as a function of the compression rate; Fitting equation, parameter and residual values for (a) and (b) can be found in Table S1 (Supporting Information); c) pore size distribution before and after calendering for different compression rates, two measurements per compaction rate have been conducted (dark and light color); d) nanoindentation behavior of SPAN, compared with popular LIB electrodes; SEM/EDS cross‐section images: e) pristine SPAN; f) SPAN with 44% compaction rate (big active material particles are encircled exemplarily).

However, the calendering behavior appears completely different for SPAN based electrodes and only minor compaction rates and density improvements are found in the cathodes after the calendering step, see **Table** **1**. Examination of the adhesion strength dependency reveals a minimum value, which is ≈93.5% of the adhesion strength of the uncalendered electrode as depicted in **Figure** **1**a. This is unexpected, as NMC cathodes with a comparable composition show a much more pronounced decrease in adhesive strength, with only ≈60–65% of the adhesion strength of uncalendered electrodes at the minimum.^[^ ^71^ ^]^ This significantly lower effect of the calender stress on the adhesive strength of SPAN cathodes points to a substantially lower rearrangement of the particles on the contact surface. Simultaneously, the resistivity measurements in Figure 1b show an exponential increase, indicating the breakdown of the percolative network within the electrode. Since SPAN has a relatively low intrinsic conductivity of 10^−3^ mS cm^−1^,^[^ ^47^ ^]^ maintaining a stable and well‐distributed network of conductive additives is crucial. The breakdown of this network is largely due to the pronounced springback behavior of the SPAN cathode. As shown in supplementary Figure S1 (Supporting Information), while the relative density achievable during calendering for SPAN cathodes is comparable to that of NMC cathodes, the springback of SPAN cathodes is considerably greater. This extensive springback is problematic as it partially undermines the benefits of calendering and requires increased effort to derive the desired density. Moreover, significant expansion of the electrode after compression in the calender gap induces additional stress on the electrode network.

**Table 1:** Obtained density and compaction rates during calendering and after calendering for the compressed and pristine electrodes.

Compaction rate in gap^a^ ^)^ [%]	Compaction rate after calendering^b^ ^)^ [%]	Density^c^ ^)^ [g cm^−3^]	Porosity^c^ ^)^ [%]
Pristine	‐	0.71	59.22
17	6	0.79	58.37
27	7	0.80	55.11
36	9	0.83	54.45
44	12	0.87	51.87
52	15	0.91	50.85
58	16	0.92	49.11
62	17	0.95	48.62

^a)^ compaction rate in gap = 1 – (real calender gap – current collector thickness) / pristine electrode coating thickness;

^b)^ compaction rate after calendering = 1 – calendered electrode coating thickness / pristine electrode coating thickness;

^c)^ from mercury porosity measurements.

The resistive compression behavior of SPAN cathodes is further evidenced by mercury porosimetry and scanning electron microscope (SEM) imaging. Only a limited variation in the pore size distribution across all calendering degrees is found, see Figure 1c. The modal pore size of the inter‐particle active material pores decreases ≈41% from 1.7 µm (uncalendered) to 1 µm (for 62% compaction rate in the gap), while, e.g., a reduction of the modal pore size of 67% from 0.45 to 0.15 µm alongside a threefold reduction in the specific pore volume was found for calendered classical LIB graphite anodes.^[^ ^75^, ^76^ ^]^ Moreover, the peak height of the inter‐particle active material pores remains largely unaffected by calendering, which is in concordance with the small increase in electrode density for increasing calendering degrees. Simultaneously, drying cracks in the range of 4–10 µm are progressively closed for increasing calendering degree while smaller pores (≤0.4 µm), which comprise of intra‐particle porosity of the active material and carbon‐binder‐domainpores, are virtually unchanged.

Energy dispersive X‐ray spectroscopy (EDS)/SEM images of the cross‐section area for an uncalendered and a calendered (44% compaction rate) SPAN cathode further visualize the morphological changes, see Figure 1e,f. Additional SEM images of the surface of the pristine and calendered electrodes and more detailed views of the cross‐sections can be found in Figure S2 (Supporting Information). Smaller surface cracks alongside a more compact packing are found for higher calendering grades, indicating a reduced macro‐pore space. This observation is in concordance with the peak shift from 1.7 to 1 µm for higher calendering grades as well as the diminished peak for pores in the range of 4–10 µm from mercury porosity results. The visibly intact SPAN particles in the cross‐section images of the calendered electrode support the unaffected intra‐particle pore space (<0.4 µm). Additionally, the aluminum current collector appears to be unharmed after compression. This suggests that solely large and easily accessible pores are affected by calendering, while the majority of the compression force is not reaching the contact area between the coating and the current collector, which is in accordance with the adhesion strength measurements.

Further insight into the probable reason for this unusual calendering behavior of SPAN cathodes can be gained by nanoindentation measurements shown in Figure 1d. The deformation behavior varies significantly among different electrode types, with graphite anodes exhibiting the most plastic behavior (median elastic work fraction of 30.6%) and SPAN and NCM cathodes being more elastic (48.2% and 61.7%, respectively). However, the deformation behavior of individual active material particles, indicated by open symbols, differs remarkably between the two cathodes. NCM particles are very brittle and plastic, while SPAN particles are as elastic as their electrodes, suggesting that SPAN electrodes have an active material dominated deformation behavior similar to graphite anodes, whereas NCM cathodes are dominated by the additive network. During calendering, the compression of the electrodes' structure will lead to a more active material dominated deformation behavior since the porosity is reduced and the active material contributes the largest volume share. So, NCM cathodes become much stiffer, while the SPAN active material is flexible, maintaining the flexibility of the electrode. This aligns with the observed large springback, the undamaged electrode structure and low relative density of calendered SPAN electrodes. Since SPAN is a polymer material, the particles are easy to compress and flexible, buffering the stress applied by the calender. Particle rearrangements occur, but not as significant as for classical LIB electrodes despite having the broad particle and pore size distributions as presented in Figure S3 (Supporting Information) and Figure 1c, which is shown to be a beneficial parameter to the fabrication of dense and homogenous LIB cathodes.^[^ ^72^, ^77^ ^]^ The calendering however results in no significant density increase in the SPAN cathode, while the electrode structure is stressed by the intense mechanical deformation during springback. Combined with a near minimal amount of carbon black, the conductive percolative network is increasingly susceptible to damage during the compression and expansion behavior, leading to a serious increase in electrode resistivity. Therefore, unlike classical LIB materials, where a broader particle size distribution enhances electrode density, the advantage of the wide particle size distribution in SPAN active material is offset by its elastic deformation behavior, leading to an inhomogeneous cathode structure.

The severity of the damage of the calendering step on the percolative network was further investigated with inline resistance measurements in dependency of the applied pressure. If the damage to the conductive network is a simple vertical contact loss, the damage could be reversible by applying vertical pressure leading to a converging of the resistance values for higher pressures. However, as depicted in Figure S4 (Supporting Information) this is not the case. The resistance of the individual electrodes decreases with increased pressure, which is expected due to the creation of more particle contacts by the electrode compression. However, an analogous behavior is found for all calendering degrees. This is an indication that the damage to the conductive network is not a simple vertical contact loss, but a complex and irreversible damage to the percolative additive network originating from the flexible active material and electrode deformation behavior leading to a springback after stress relief. Increasing the amount of conductive agent would possibly alleviate the damages toward the percolation network after calendering. However, practical electrode design requires maximizing the gravimetric capacity and optimizing the ratio of active to passive materials.

**Figure 2:**
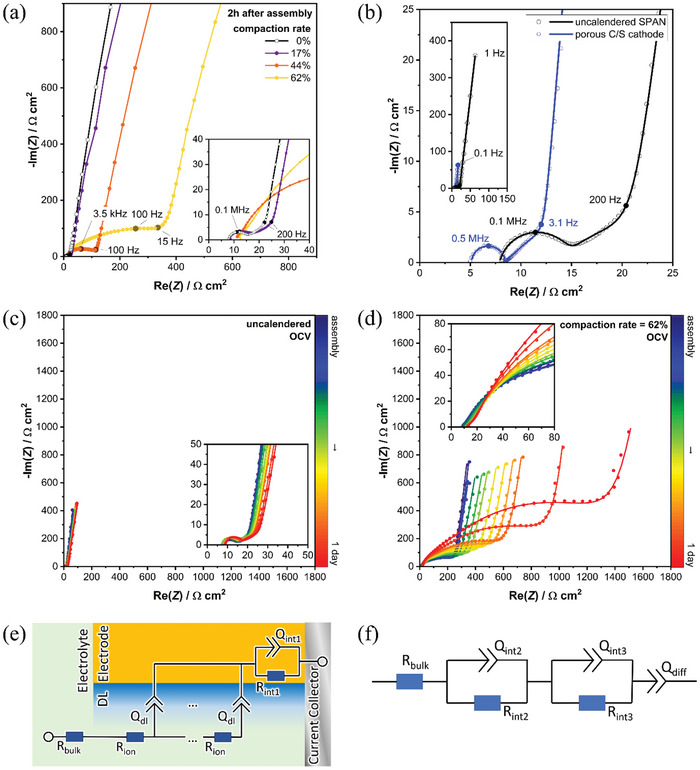
Nyquist presentation of symmetrical OCV impedance data of a) four different calendering grades after 2 h during OCV; b) SPAN in comparison to an ideal porous carbon/sulfur (C/S) composite electrode; OCV impedance behavior over one day for c) uncalendered SPAN cathode and d) SPAN cathode with 62% compaction rate; equivalent models for fitting of e) porous cathodes (TLM) and f) affected SPAN cathodes (R‐RQ‐RQ‐Q).

In order to elucidate the effect of calendering on the microstructure of the cathode and the measured increased electrode resistances, further investigations were carried out using electrochemical impedance spectroscopy. **Figure** **2**a shows the open circuit voltage (OCV) impedance spectra of symmetrical cathode/cathode cells of various calendering grades two hours post‐assembly. For the uncalendered electrode and the electrode with minor compaction rate (17%), a small process in the high frequency area followed by a straight contribution with ≈45° incline in the mid‐frequency range can be found. Meanwhile, medium and high calendering grades possess a big and distorted semicircle contribution in the high‐ to mid‐frequency area. In the low‐frequency regime, all calendering grades show the characteristic blocking conditions resulting from the insulating properties of sulfur, which are shown in Nyquist presentation as a straight and steep branch.^[^ ^78^, ^79^ ^]^ Moreover, while for small or non‐calendering grade electrodes a virtually stable impedance signal is found over the whole measurement time, the high‐ to mid‐frequency semicircle keeps growing for electrodes with greater calendering grade, see Figure 2c,d and Figure S5 (Supporting Information).

The impedance response of the uncalendered and low‐calendered SPAN cathodes shows analogy to reference porous carbon/sulfur composite cathodes, as visible in Figure 2b. Herein, the semicircle is associated with inter‐particle effects (RQ_int_, inter‐particle effects within the cathode as well as the contact with current collector) while the 45° incline depicts the ionic resistance in the porous system (R_ion_) of the composite electrode.^[^ ^79^ ^]^ Although the characteristic shape of impedance signals for porous materials remains identifiable for uncalendered and low‐calendered SPAN, it appears slightly distorted. However, higher calendering grades show a complete lack of porous response whereby the designative features seem to be superimposed by other contributions. As discussed, the interconnecting electronic pathways of the SPAN cathodes may be partly destroyed with growing compression grade, thus facilitating inhomogeneous conductive regions within the electrode. Regions of different morphology and electronic conductivity hold various time constants and arise as multiple and overlapping effects in the impedance response.^[^ ^80^ ^]^

While regular porous impedance features are typically fitted with a transmission line model (TLM),^[^ ^81^ ^]^ see Figure 2e, a simplified method must be used for more intensely compressed SPAN cathodes to take account for the inhomogeneity in the SPANs electronic network. Therefore, the equivalent circuit displayed in Figure 2f is applied for fitting. The serial resistance element R_bulk_, which is mostly influenced by the electrolyte bulk resistance, is followed by two parallel RQ‐elements representing features in the high‐ and mid‐frequency region. The imaginary characteristics for these processes are modeled with the constant phase element Q. An additional constant phase element Q_diff_ is added serially in order to reproduce the cells' blocking behavior while taking account of small non‐ideality features due to slight diffusive activities in the solid cathode material.^[^ ^82^ ^]^ It is essential to acknowledge that results obtained by such simplified equivalent models contain inaccuracies, yet it allows for a qualitative analysis of the detected effects. To distinguish the different co‐occuring inter‐particle effects, the time constants have been calculated, given in Section S3 (Supporting Information). Inter‐particle resistances occurring at high frequencies are denoted as R_int1_. For uncalendered cathodes as well as for cathodes with low calendering grade, R_ion_ represents the impaired pore‐resistance, affected by inter‐particle effects of medium relaxation times and thus appearing in a slightly distorted manner. However, for highly compressed electrodes the porous characteristic is completely superimposed by the dominant responses of R_int2_ and R_int3_, exhibiting the deteriorated inter‐particle electronic network regimes, which possess medium or short relaxation times.

**Figure 3:**
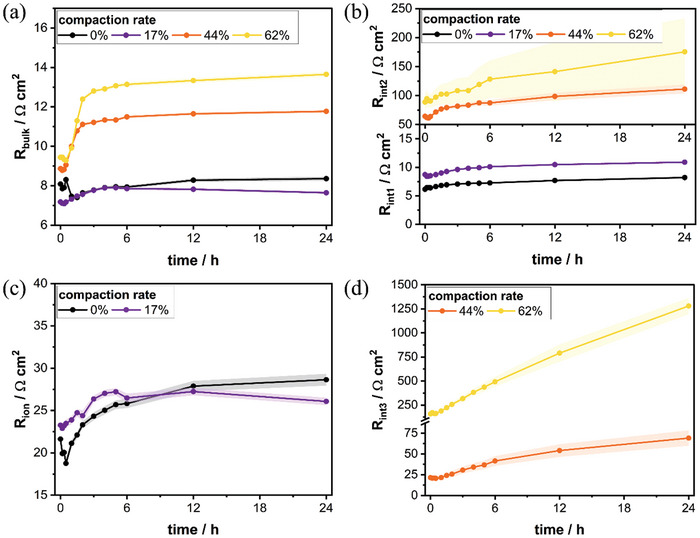
Fitted resistance contributions of the uncalendered and calendered cathodes using an TML or R‐RQ‐RQ‐Q equivalent model; a) bulk electrolyte resistance R_bulk_, b) inter‐particle resistance in high‐frequency regime P2, c) pore resistance, d) inter‐particle resistance in mid‐frequency regime.

**Figure** **3**a shows the development of the bulk electrolyte resistance R_bulk_ for all four calendering grades over the course of time after electrolyte filling. While after initial wetting fluctuations the series resistance is relatively constant at 7.7 to 8.3 Ω cm^2^ for the uncalendered electrode and electrode with 17% compression rate, calendering seems to have a gradual impact and the resistance rises up to 13.6 Ω cm^2^ for the cathode with the highest compression. This is in concordance with the similar trends found for the measured volume and two‐point resistivity, see Figure 1b, indicating that the compression‐induced degradation of the conductive network is appearing throughout the electrode. Moreover, high compression grade cathodes exhibit a growing electrolyte resistance in the first 5 h,‐ indicating an increased ohmic contribution of the electrode resistance during wetting. While the high‐frequency regime inter‐particle resistance R_int1_ shows stable values ≈8.2 to 10.9 Ω cm^2^, increased values more than one order of magnitude higher are found for R_int2_ for higher calendering degrees, see Figure 3b, confirming the negative impact of the cathode compression toward the electronic network. However, only a small influence toward the porous feature is found, see Figure 3c, and resistance values are stabilizing ≈26 to 29 Ω cm^2^ for both, the uncalendered electrode and the 17% compaction rate cathode. The lowfrequency inter‐particle contribution R_int3_ arising from locally stronger damaged areas due to more intense compression is depicted in Figure 3d. While for the medium calendered electrode with 44% compaction rate this influence is ≈21 Ω cm^2^ after cell assembly, the 62% compaction rate cell shows an almost tenfold magnitude. Moreover, steady growth can be monitored over the progression of time, leading to excessive resistances up to 1280 Ω cm^2^ after 12 h in OCV. The increasing values may arise due to swelling of the cathode, leading to even greater destruction of the electronically conducting pathways in the already damaged areas.

**Figure 4:**
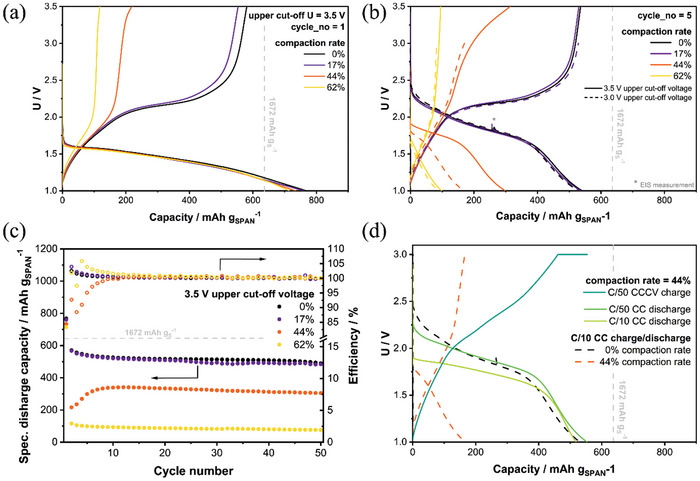
Cycling data of cathodes with various compression rates: a) 1st cycle with 3.5 V upper cut‐off potential; b) comparison of 3.0 and 3.5 V upper cut‐off potential; c) capacity and coulombic efficiency of 3.5 V cells; d) effect of C‐rate toward voltage plateaus and capacity of a medium compressed electrode.

For further investigations the prepared and morphologically characterized cathodes have been assembled with a lithium metal anode and cycled at different C‐rates as well as a different voltage range. **Figure** **4**a depicts the first discharge/charge cycle for uncalendered and calendered samples cycled between 1.0 to 3.5 V whereby no compression‐induced impact can be found for the first discharge cycle. The discharge plateaus as well as specific capacities are identical for all the cathodes. During the first reduction step, lithiation and molecular rearrangement of the cathode's polymeric backbone occur besides Li_2_S formation, which inherits a low discharge potential plateau at ≈1.6 V and a higher irreversible capacity, observed frequently within SPAN studies.^[^ ^28^, ^30^, ^83^ ^]^ However, the following charge process appears to be substantially different for the different calendering rates, whereby the charge capacity decreases from 530 to 100 mAh g_SPAN_^−1^ (1400 to 250 mAh g_S_^−1^) for the uncalendered and highest compression grade cathode, respectively. Figure 4b presents the voltage profiles of the following fifth cycle (solid lines). For cathodes with a small compression grade, where the first charge is not highly impacted, no decisive effect on the following discharge/charge plateaus can be noticed and the cells exhibit stable cycling performance with the anticipated capacity of 530 mAh g_SPAN_^−1^, see Figure 4c. For medium grade compression (44% compaction rate), a capacity decline toward 290 mAh g_SPAN_^−1^ (750 mAh g_S_^−1^) occurs. Moreover, the charge plateau is shifted notably by ≥1.0 V toward higher voltage regimes, while the discharge plateau is lowered by roughly 0.4 V (at the same C‐rate). Therefore, the upper cut‐off voltage was elevated from the typical 3.0 V (dashed lines) to 3.5 V (solid lines) in order to reach the main charge plateau for the medium compressed SPAN cells at all, while it is completely cut off for highly compressed cathodes. The reduced capacity output for compressed cathodes mainly stems from increased overpotential upon charge, which is causing the shift in the voltage plateau. Thereby, the cut‐off voltage is reached before the completion of charging process and a reduced charge capacity is obtained already in the first charge process, despite the unaffected first discharge. The uplift trend of the charge voltage plateau and the effect on the capacity can also be observed in further compaction rates, see Figure S6 (Supporting Information).

The elevated charge plateau leads to incomplete utilization of the active material and thus is limiting the reduction capability in the following discharge. As a consequence, less capacity can be obtained for medium calendering grades cycled up to 3.0 V, see Figure S7 (Supporting Information). In fact, rising the cut‐off voltage up to 3.5 V provides a capacity gain of ≈100% for the 44% compaction rate calendered electrode. For high calendering grades (62% compaction rate) the charge plateau still cannot be reached due to excessive overpotential and increasing the upper cut‐off potential is limited by the electrolyte's stability window.^[^ ^84^ ^]^ The premature termination of the charge process yields a marginal capacity of ≈100 mAh g_SPAN_^−1^ (250 mAh g_S_^−1^), yet showing reproducible cycling behavior. This remaining capacity regardless of the calendering degree stems either from the backbone or from C‐S‐Li parts of the discharged cell, where one remaining sulfur atom stays attached to the polymeric backbone,^[^ ^33^ ^]^ which still can be lithiated during discharge. Due to the covalent bonding, an improved electronic connection to the backbone is given, compared to Li_2_S crystals in the pores. This corroborates with the suggested charging mechanism possessing two‐step behavior with its dominant process of Li_2_S oxidation arising at >2 V, affected by calendering and resulting in a plateau shift, and an inferior oxidation process at 1.0 to 2.0 V that is unaffected by calendering.

In order to understand why this effect is pronounced upon charge while not affecting the first discharge, the 44% compression rate cathode has been discharged at different C‐rates while charging was performed at C/50 and a constant voltage (CV) step was added to the charging process (Figure 4d, blue solid line), insuring a fully charged cathode. The upper cut‐off voltage was again set to typically used 3.0 V, as narrower cut‐off voltages tend to enhance battery cyclability and fully charging of the affected compressed cathodes is enforced by the CV step.^[^ ^85^ ^]^ The previously discussed uncompressed cathode and cathode with 44% compression rate cycled at C/10 in between 1.0 to 3.0 V was added to the graphic (dashed lines) for comparison.

Full discharge capacity can be regained for compressed cathodes, if previous charging was complemented by a CV step, independently from the C‐rate (yellow solid line), confirming that the active material remains intact during calendering. The discharge plateau is still lowered by 0.3 to 0.4 V compared to the uncalendered electrode of the same C‐rate. If the C‐rate of the compressed electrode is lowered to C/50 (green solid line) no decisive impact on the discharge plateau is observed anymore. However, the charge plateau (blue solid line) still shows enlarged overpotentials, although less intense, indicating the charge limitations observed for compressed cathodes cycled at reasonable current densities are mainly stemming from transport limitations. Moreover, the fully accessible discharge capacity for fully charged cathodes (green and yellow solid lines) as well as the intact first discharge cycle for all calendering degrees rules out hindered Li‐ion diffusion or wetting issues due to pore‐space restrictions. The augmented impact of cathode compression on the cell's charging capabilities is further highlighted through discharge and charge C‐rate tests, as shown in Figure S8 (Supporting Information). Across all compression rates, the specific discharge capacity exhibits a greater reduction during charge rate tests (varying charging current while maintaining a constant discharge current) compared to discharge rate tests. As an increase of current density leads to greater overpotentials, the observed premature termination of the charge process due to the higher charge overpotentials is amplified. However, subsequent long‐term cycling at moderate current densities for 50 cycles shows stable behavior for all compression rates, emphasizing that the origin of the decreased capacity is not due to mechanically damaged active material but kinetic or transport limitations.

As the prepared SPAN cathodes consist of an already close to minimum percentage of conductive agent while the deployed SPAN particles exhibit a broadened particle size distribution accompanied by a pronounced springback effect after compression, calendering promotes inhomogeneities in the cathodes morphological structure, i.e., the partial disturbance of electronic conduction pathways.^[^ ^86^ ^]^ We propose that the kinetic domination of Li_2_S oxidation accompanied by the damaged electronic conductive pathways are the main reasons for increased overpotentials upon charge.

In order to understand the influence of heterogeneity of ionic and electronic conduction pathways on the impedance response and accessible capacity, we have conducted a series of simulation studies presented followingly.

**Figure 5:**
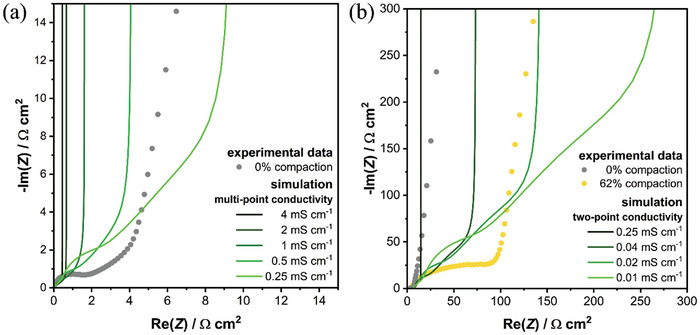
Effect of varying homogeneous electric conductivity on impedance response of symmetric cell at OCV. Solid lines represent simulated spectra using the continuum model. Circles indicate experimental data of a) the uncalendered electrode (grey circles) and b) highly calendered electrode (62% compression rate, yellow circles). Real part corrected by high frequency intersect. a) Conductivity determined by multi‐point measurements in the electrode plane. b) Conductivity range determined by two‐point measurements.

In a first step we simulate the impedance response of uncalendered and highly calendered (62% compaction rate) SPAN electrodes in symmetric configuration at OCV. **Figure** **5** shows simulated impedance spectra of the uncalendered electrodes. The effective conductivity is varied in the range observed in electronic resistance measurements for different calendering grades. Solid lines represent corresponding simulation results with homogeneous conductivity of the conductive network and circles show experimental data measured on uncalendered electrodes after assembly. Note, that the simulations with lowest electronic resistance, as measured by the multi‐point method on uncalendered samples, shows significantly lower electrode impedance compared to the EIS data. This indicates that the in‐plane conductivity is significantly higher compared to the through‐plane conductivity measured with two‐point probe measurements. With increasing resistance, the pore transport contribution to the impedance, indicated by the linear increase of the imaginary part in the Nyquist plot, consistently increases. This confirms that the conductive network in the uncalendered electrodes is rather homogeneous. At a simulated electronic resistance between 2 and 4 kΩ cm (0.5–0.25 mS cm^−1^) we observe good qualitative agreement with the experimental data. Note, that given the previous discussions we focus in our analysis on the effect of electronic transport networks. Results on the variation of effective ionic conductivity are provided in Figure S9 (Supporting Information).

The electrode's electrical conductivity determined by multi‐point measurements (Figures 1b and 5a) is still orders of magnitude higher compared to the results of the two‐point measurements. Figure 5b shows additional impedance data of simulations with electrode resistance between 4 and 100 kΩ cm (0.25 to 0.01 mS cm^−1^). Despite significantly increasing the electrode resistance, the impedance response does not show the characteristic semi‐circle observed for high calendering grades. Therefore, we propose that the calendering introduces structural heterogeneity unfavorably affecting the electronic and ionic transport networks.

**Figure 6:**
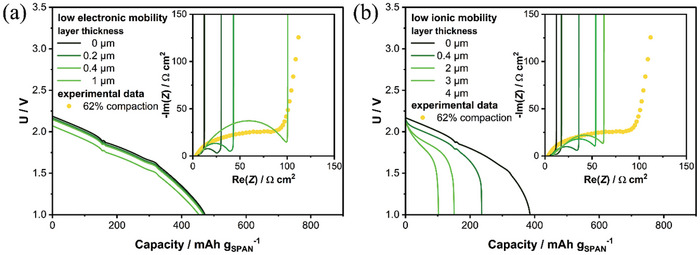
Impact of heterogeneity in conductive networks on discharge performance at C/3. Colors indicate layers with a) low electronic (0.001 mS cm^−1^) and b) ionic (β  =  10) mobility. The thickness of the layers is varied between 0 and 4 µm. The inset shows the corresponding impedance simulations.

Following up on our study with homogeneous conductive networks, we introduce inhomogeneities in the form of layers with low electronic conductivity of SPAN close to the current collector. This approach essentially describes the breakdown of a percolating network due to the springback effect. The inset in **Figure** **6**a shows impedance spectra simulated using electrode parameters of a highly calendered electrode with increasing layer thickness of the non‐conductive layer. The corresponding experimental data is included as circles in the graph. The simulated spectra qualitatively reproduce measured impedance spectra, indicating that heterogeneities in the conductive network arise due to calendering. Note, that similar effects can be a result of dense layers close to the separator, which blocks ion transport. Dense layers can be expected due to compaction and plastic deformation of SPAN particles at high calendering rates. Therefore, we additionally simulate increasing dense layers with low ion mobility. Impedance spectra are presented as inset in Figure 6b. Indeed, we observe similar features, however even thick layers are not able to reproduce the measured impedance spectra, indicating that electronic transport effects are dominating the impedance spectra.

Additionally, to conclude our simulation study we perform discharge simulations at C/3 rate to highlight the effect of inhomogeneous conductive networks, both electronic and ionic, on the electrode performance. Figure 6a shows the effect of increasing layers with low conductivity representing the case with damaged electronic percolating network. In all cases the resistive layer decreases the cell voltage, however, does not have a major impact on the discharge capacity corroborating our experimental data recorded with low charging currents and an additional CV step, see Figure 4d. The resistive layer increases overpotentials resulting in a linear shift of the cell voltage. Note, that the resistive layer does not change the slope of the upper plateau in the discharge curve. Figure 6b shows the complimentary study investigating heterogeneous ion transport. In contrast a dense layer reducing ion mobility significantly reduces discharge capacity and additionally results in a steeper slope of the upper plateau.

This indicates that transport limitations due to calendering, either electronic or ionic, result in a negative feedback on cell capacity. However, as mentioned above this result is enhanced by slow charging kinetics. Analysis of the upper plateau of experimental discharge curves shows that the slope is changing only marginally, despite significant loss in capacity. Therefore, we conclude that ionic transport limitations cannot be excluded, however, as indicated by the impedance data and electrode characterization the loss of a homogeneous electronic network is the main contributor to the decreasing electrode performance.

## 3 Conclusion

SPAN cathodes were manufactured and calendered with varied compression grades. Even for high calendering grades, limited density increases were found within the cathodes. Nanoindentation tests exhibited a highly elastic deformation behavior of both, the SPAN active material particles as well as the cathodes, suggesting a pronounced springback and an active material particle dominated deformation behavior of the electrodes, which might be transferable to other organosulfur cathode materials with similar characteristics. The SPAN cathode's elastic characteristics result in a deficient percolative carbon black network, which seems to be destroyed during springback. Irreversible conductivity degradation was also found in electronic conductivity measurements, which showed increased resistances for higher compression grades, which is in corroboration with electrochemical impedance data on symmetrical cells. The impedance response of the highly calendered samples does not exhibit the porous characteristics of sulfur cathodes. Instead, high impedance inter‐particle processes with different time constants are identified, reflecting the areas of varying electronic conductivity within the inhomogeneous electrode. These morphological changes within the cathode upon calendering negatively impact the cell capacity due to transport limitations, whether electronic or ionic. Our experimental electrochemical data – showing full capacity in the first discharge, significant capacity loss during the following charge, minimal changes in the upper plateau of discharge curves, and recoverable capacity at slower C‐rates – corroborate our simulation data, which conclude that this phenomenon is primarily due to the loss of a homogeneous electronic network rather than ionic conduction limitations. Based on this finding we propose fabricating reliable, densified SPAN electrodes by ensuring uniform structure, increasing material stiffness and plasticity, improving the binder system for better compaction, and using conductive additives like carbon nanotubes, graphene, or carbon nanofibers to maintain the percolation network and reduce springback. An intact conductive network and minimized springback are essential for successful SPAN cathode compaction.

## Conflict of Interest

The authors declare no competing interests.

## Supporting information



Supporting Information

## Data Availability

The data that support the findings of this study are available from the corresponding author upon reasonable request.
