# Supplementary material for: Impact of the Sulfurized Polyacrylonitrile Cathode Microstructure on the Electrochemical Performance of Lithium–Sulfur Batteries
Source: Adv Sci (Weinh). 2025 Feb 22;12(15):2415436. doi: 10.1002/advs.202415436 (PMC12005800; doi:10.1002/advs.202415436)
Supplement: Supplementary file 1 — Supporting Information [file ADVS-12-2415436-s001.docx]

Supporting Information

Impact of the Sulfurized Polyacrylonitrile Cathode Microstructure on the Electrochemical Performance of Lithium-Sulfur Batteries

*Robin Moschner*^,ⱡ,1^, Martina Gerle^ⱡ,2^, Timo Danner^2,3^, Esther Kezia Simanjuntak^2,3^, Peter Michalowski^1^, Arnulf Latz^2,3,4^, Maryam Nojabaee*^,2^, Arno Kwade^1^, K. A. Friedrich^2,5^*

*[r.moschner@tu-braunschweig.de](mailto:r.moschner@tu-braunschweig.de)

*[maryam.nojabaee@dlr.de](mailto:maryam.nojabaee@dlr.de)

^1^ Institute for Particle Technology, Technische Universität Braunschweig, Volkmaroder Straße 5, D-38104 Braunschweig, Germany

^2^ Institute of Engineering Thermodynamics, German Aerospace Center (DLR), Pfaffenwaldring 38-40, D-70569, Stuttgart, Germany

^3^ Helmholtz Institute Ulm (HIU) Electrochemical Energy Storage, D-89081, Ulm, Germany

^4^ Institute of Electrochemistry, Ulm University, D-89081, Ulm, Germany

^5^ Institute of Energy Storage, University of Stuttgart, Pfaffenwaldring 31, D-70569 Stuttgart, Germany

Keywords: Lithium-sulfur battery, sulfurized polyacrylonitrile, calendering, mechanical characterization, electrochemical impedance spectroscopy, electrochemical characterization, numerical simulation

ⱡ Robin Moschner and Martina Gerle contributed equally.

# Experimental Section

SPAN electrodes with 3.5 mAh cm^-2^ nominal capacity were produced, comprising of 90 wt.-% of commercially available SPAN powder (ADEKA CORPORATION, ADEKA AMERANSA SAM-1, 38 wt.-% sulfur), 5 wt.-% Super C65 carbon black (TIMCAL Ltd.), 3 wt.-% styrene butadiene rubber (SBR, ZEON CORPORATION, BM-451B, pre-dispersed) and 2 wt.-% carboxymethyl cellulose (CMC, DuPont de Nemours, Texturecel 2000 PA09). The amount of conductive agent was set to a minimum value to increase industrial relevance and amplify influences on the electrode properties by the production process to achieve clear results. An aqueous slurry was fabricated using a dissolver (AE04, VMA-GETZMANN GMBH) with a tip speed of 3 m s^-1^ after active material addition with a target solids content of 40 wt.-%. The cathode ink was further processed in a pilot scale roll-to-roll coating and drying machine (LabCo, KROENERT GmbH & Co KG), using a comma-bar-reverse-roll coating approach and convective drying at 60 °C as described in more detail in our previous study. ^[1]^ As current collector, a 15 µm uncoated aluminum foil A1100-L was used and the dry coating thickness (without current collector) was measured to be 102 µm ± 3 µm.

For the NCM cathode used as reference in this paper a composition of 95 wt.-% of NCM622 by BASF was used together with 1.75 wt.-% Super C65 carbon black by TIMCAL Ltd., 1 wt.-% of Timrex SFG6L conductive graphite by TIMCAL Ltd. and 2.25 wt.-% of Solef PVdF 5130 by Solvay. The cathode was produced using the same dissolver AE04 by VMA-GETZMANN. All solid components were dry mixed with low intensity before adding them to NMP with a target solids content of 70 wt.-%. The slurry was mixed using a 50 mm toothed disk at 9 m/s tip speed for 30 minutes and was degassed in the last 15 minutes of this time at 200 mbar total pressure. The slurry was then coated with the described pilot coater LabCo onto uncoated aluminum foil as well, using a drying setup with three drying sections at 80, 100 and 120 °C respectively. The 104 µm ± 4 µm uncalendered dry coating thickness was set to be the same as for the SPAN cathode without current collector.

The graphite anode was produced using 95 wt.-% of artificial graphite, 1 wt.-% of Super C65, 2.67 wt.-% of BM-451B SBR binder (pre-dispersed) and 1.33 wt.-% of CMC Texturecel 2000 PA09 as used for the SPAN cathodes as well. All components except the pre-dispersed SBR were dry mixed with low intensity and then added to deionized water with a target solids content of 47.5 wt.-%. The slurry was then mixed for 10 minutes at 9 m/s tip speed also using a 50 mm toothed disk. Next the SBR solution was added and the slurry was again mixed for 15 minutes at 3 m/s tip speed while it was degassed at 200 mbar total pressure. The finished slurry was coated onto a 10 µm copper foil with a dry coating thickness of 102 µm ± 2 µm. The graphite anode was dried at 60 °C like the SPAN cathode.

The carbon/sulfur reference cathode was prepared using 63 wt.-% of a meso-porous carbon (Ketjenblack KB EC-600 JD, Akzo Nobel), 27 wt.-% of sulfur (Alfa Aesar), 3 wt.-% CMC (Walocel CRT 2000 PA, Dow Wolff) binder and 7 wt.-% PEO (polyethylene oxide, Sigma Aldrich, Mv = 600 000) binder. Sulfur and carbon were dry-mixed in a ball-milling approach at 700 rpm for 15 minutes before being added to the pre-dispersed aqueous binder solution, aiming for 11 % solid fraction. The slurry was mixed in a tumbling-shaker for 8 hours and coated by doctor blading approach on a 23 µm thick carbon-primed aluminum foil.

For calendering, a pilot scale calender (GKL400 MS, SAUERESSIG Group) with a maximum line load of 1500 N mm^-1^ was used to compress the electrodes by adjustment of the calender gap. The nominal calendering gap values were set to 100, 85, 70, 55, 40, 25 and 10 µm. Measurements of the true gap height were obtained using capaNCDT sensors (Micro-Epsilon Messtechnik GmbH & Co. KG) as described by Diener et al.^[2]^ The final electrode thickness after calendering was tracked using an absolute digimatic indicator (Mitutoyo Corporation). Pore size distribution measurements were obtained using mercury intrusion porosimetry (PoreMaster 60GT, Anton Paar Germany GmbH) with 40 cm² sample size in a pressure range of 10 to 30.000 PSI, which allows the characterization of pore sizes in the range of 7 nm to 21 µm. The procedure is described in detail by Froboese et al.^[3]^ Two measurements per calendering degree were performed.

Scanning electron microscope (SEM) images were recorded using a Zeiss Crossbeam 350 field emission SEM equipped with energy dispersive X-ray spectroscopy (EDS) detector from Oxford (ULTIM MAX 100 mm^2^) for elemental analysis. An ion cutting system from JEOL (IB-19520/CCP) was used with Argon gas at 6 kV for 10 h to prepare the cross-sections. The samples were cooled with liquid nitrogen during cutting operation and sandwiched between two Si-wafers without embedding resin.

Particle size distributions of the pristine AM-powders were measured using the Partica LA-960 laser diffraction device by Horiba Scientific with a laser wavelength of 650 nm. For sample deagglomeration compressed air was used while the sample was fed to the measurement device by a vibrating ramp. The particle size distributions were derived by combining at least 7 measurements each using diffraction indices of 1.69 – 0.24i, 1.50 – 0.10i and 1.70 – 0.05i for SPAN, NCM 622 and Graphite, respectively.

Nanoindentation experiments on the active material particles were conducted for insights into the particle’s elastic-plastic behavior, using a Hysitron TI 950 Nanoindenter (Bruker Corporation). The particles were compressed using a 50 µm flat punch geometry with an indentation depth of about 10% of their total approximated size. A minimum of 40 particles was measured per sample to achieve reliable values. Nanoindentation measurements of the electrodes were performed using a UNAT Indenter by ASMEC GmbH and a 100 µm flat punch geometry. Each sample was indented at least 50 times. The indentation depth was set to 10% of the electrodes coating thickness to exclude any influence of the collector foil on the deformation behavior.

To characterize the adhesion strength, 90° pull-off tests were conducted, as described in detail by Haselrieder et al.^[4]^ Samples with an area of 1.13 cm² were pressed by the material testing machine Z020 by ZwickRoell GmbH & Co. KG with 0.6 MPa for 30 s. Subsequently, the upper geometry is pulled off at a speed of 100 mm min^-1^, and the maximum force required is measured at 2000 Hz. Five individual measurements were carried out for each calendering degree.

The conductivity of SPAN electrodes with various calendering degrees was characterized using a two-point measurement technique at the material testing machine Z020 by ZwickRoell GmbH & Co. KG. During the measurement the electrode is pressed between two stamps and a constant current is applied. The sample resistivity is calculated using the measured voltage drop, along with the sample’s area and thickness. The method is best described by Westphal et al.^[5]^ Ten measurements were carried out per calendering degree. Expanding this method, the electrodes were also compressed to different end-pressures of 3.5, 10, 20, 40 and 60 bar and then relieved, (both at 0.75 mm min^-1^), while the electronic resistance of the electrode was measured inline, allowing for a pressure-resistance correlation. The applied currents for the electrodes with various calendering degrees are displayed in **Table S 2**. Each sample and maximum force were reproduced five times. In addition, the electronic resistivity of the electrodes was measured with a multipoint surface method ten times per sample using an electrode resistance measurement system (RM2610, HIOKI E.E. CORPORATION).

For electrochemical characterization, laboratory cells (ECC-PAT-Core, EL-CELL) were assembled in an argon-filled glovebox (GS Glovebox Systemtechnik, <1 ppm H_2_O, < 1 ppm O_2_). Disks of 18 mm diameter were punched out of the cathode coatings and transferred to the glovebox via heat treatment at 120 °C and -1 bar for 14 hours. Pure lithium (500 µm, MSE Supplies, BR0146, 99.9%) was used as counter electrode and Celgard 2500 membrane as separator. The electrolyte was a 0.625 M LiNO_3_ (lithium nitrate, Sigma-Aldrich, 229741, 99.99%) + 0.375 M LiTFSi (bis(trifluormethylsulfonyl)imide, Sigma-Aldrich, 544094, 99.95%) in G2 (bis(2-methoxyethyl)ether, diglyme, Thermo Scientific, 443101000, ≥99%) which was poured on the separator with an amount of 13 ml g_S_^-1^. Galvanostatic cycling measurements were performed on a BaSyTec CTS Lab setup in between 1 to 3 V. For the combined C-rate and long-term cycling tests, CR2032 coin cells with 3.5 mAh cm^-2^ cathodes and 50-60 µl electrolyte were built and cycled without CV step as described in detail in Moschner *et al*. ^[1]^.

For electrochemical impedance spectroscopy (EIS) measurements at open circuit voltage (OCV), a symmetrical cell set-up with two cathodes was utilized. The EIS measurements were recorded in potentiostatic mode with a 10 mV excitation signal in between a frequency range of 4 MHz to 1 Hz over a time span of 24 hours, using a Zennium pro workstation (Zahner-Elektrik). The specific measurement time steps are listed in **Table S 3.**

In order to support experimental analysis, we performed electrochemical simulations on the continuum scale. The model derivation and implementation are reported in detail in ^[6]^. The model is used to simulate both discharge curves and electrochemical impedance spectra. Model parameters represent the experimental setup described in the previous paragraph and are reported in **Table S 4** to **Table S 6.** Specifically, we simulate impedance spectra in symmetrical cells at OCV with high spatial resolution at the interfaces towards current collector and separator to resolve heterogeneities in conductive networks.

1. **Supplementary data**


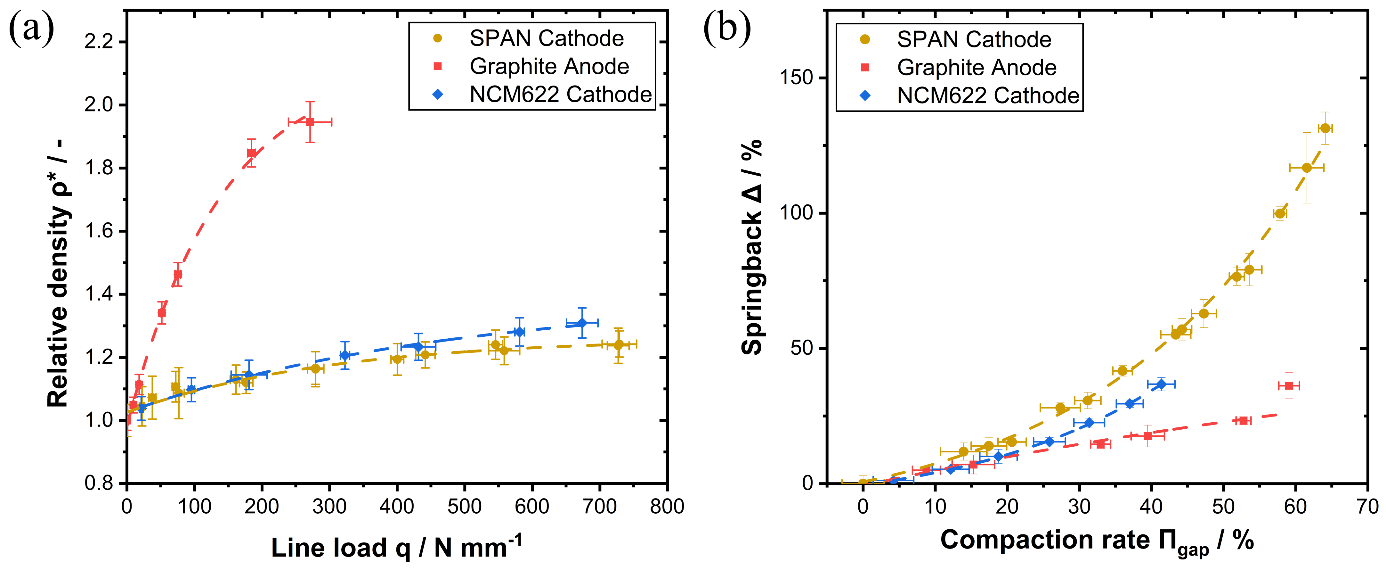


**Figure S 1.** a) Densification of electrodes right after calendering, the relative density is calculated using ρ* = ρ_calendered_ / ρ_pristine_; b) springback of electrodes right after calendering, springback is calculated using Δ = (d_after_ - d_gap_) / d_gap_ and compaction rate using Π_gap_ = (d_pristine_ - d_gap_) / d_pristine_, whereby d is the electrode thickness including the current collector foil. Fitting equations, parameter and residual values can be found in **Table S 1**.

**Table S 1.** Fitting equations, parameter and residual values

| **Figure** | **Curve** | **Equation** | **Fitting Parameter** | **R-Square (COD)** | **Adj. R-Square** |
| --- | --- | --- | --- | --- | --- |
| Figure 1 (a) | Red, dashed | $y=A+Bx+Cx^{2}$ | A = 918.98583 ± 17.10789  B = -4.8571 ± 1.9096  C = 0.09833 ± 0.01686 | 0.94208 | 0.91891 |
| Figure 1 (b) | Black, dashed | $y=A_{1}*\exp\left( -\frac{x}{t_{1}} \right)+ y_{0}$ | y_0_ = 44.1541 ± 19.52549  A_1_ = 39.4487 ± 13.49193  t_1_ = -15.94157 ± 1.59343 | 0.98118 | 0.97366 |
| Figure S 1 (a) | Red, dashed | $y=A_{1}*\exp\left( -\frac{x}{t_{1}} \right)+ y_{0}$ | y_0_ = 2.13769 ± 0.06103  A_1_ = -1.15638 ± 0.05765  t_1_ = 139.6164 ± 14.0004 | 0.99812 | 0.99718 |
|  | Blue, dashed | $y=A_{1}*\exp\left( -\frac{x}{t_{1}} \right)+ y_{0}$ | y_0_ = 1.39481 ± 0.03566  A_1_ = -0.36801 ± 0.03228  t_1_ = 488.8276 ± 88.4210 | 0.99661 | 0.99491 |
|  | Yellow, dashed | $y=A_{1}*\exp\left( -\frac{x}{t_{1}} \right)+ y_{0}$ | y_0_ = 1.26196 ± 0.01703  A_1_ = -0.23628 ± 0.01559  t_1_ = 299.2408 ± 56.8934 | 0.96682 | 0.9613 |
| Figure S 1 (b) | Red, dashed | $y=A_{1}*\exp\left( -\frac{x}{t_{1}} \right)+ y_{0}$ | y_0_ = 66.29883 ± 67.34404  A_1_ = -67.01097 ± 66.6671  t_1_ = 115.7615 ± 145.6231 | 0.96537 | 0.94805 |
|  | Blue, dashed | $y=A_{1}*\exp\left( -\frac{x}{t_{1}} \right)+ y_{0}$ | y_0_ = -10.90975 ± 1.65139  A_1_ = 10.37121 ± 1.37572  t_1_ = -27.12212 ± 1.85176 | 0.99954 | 0.9993 |
|  | Yellow, dashed | $y=A_{1}*\exp\left( -\frac{x}{t_{1}} \right)+ y_{0}$ | y_0_ = -16.56652 ± 3.94281  A_1_ = 17.20583 ± 2.6323  t_1_ = -30.28111 ± 1.97872 | 0.99641 | 0.99581 |


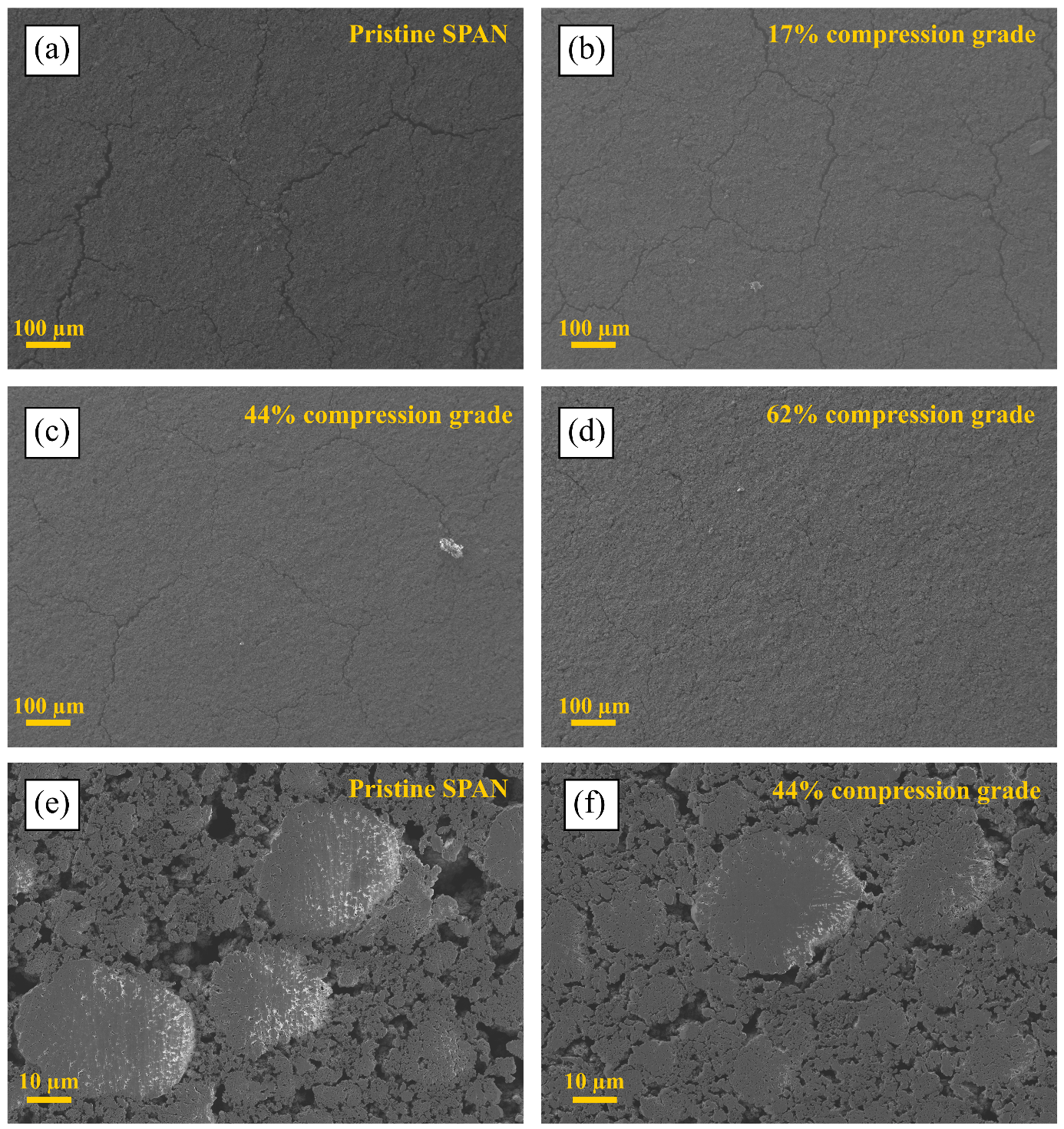


**Figure S 2.** SEM images a) surface of uncalendered/pristine SPAN cathode; b) surface of SPAN cathode with 17% compaction grade; c) surface of SPAN cathode with 44% compaction grade; d) surface of SPAN cathode with 62% compaction grade; e) cross-section area of uncalendered electrode; f) cross-section area of electrode with 42% compaction grade

**Table S 2.** Resistance ranges and applied measurement currents for the different calendering degrees of the SPAN cathodes

| Nominal calender gap [µm] | Compaction rate in gap [%] | Resistance range [kΩ] | Applied measurement current [mA] |
| --- | --- | --- | --- |
| Pristine | 0 | < 0.2 | 50 |
| 100 | 17 | < 0.2 | 50 |
| 85 | 27 | < 2 | 5 |
| 70 | 36 | < 2 | 5 |
| 55 | 44 | < 2 | 5 |
| 40 | 52 | < 2 | 5 |
| 25 | 58 | < 20 | 0.5 |
| 10 | 62 | < 20 | 0.5 |

**Table S 3**. Time intervals of impedance measurements during OCV

| Measurement No. | Time after assembly |
| --- | --- |
| 1 | - |
| 2 | 10 min |
| 3 | 20 min |
| 4 | 30 min |
| 5 | 1 h |
| 6 | 1.5 h |
| 7 | 2 h |
| 8 | 3 h |
| 9 | 4 h |
| 10 | 5 h |
| 11 | 6 h |
| 12 | 12 h |
| 13 | 24 h |

**Table S 4.** Geometrical parameter of uncalendered electrode and uncalendered electrode with 10 µm calender gap.

|  | **SPAN electrode** | |
| --- | --- | --- |
|  | **Uncalendered** | **Calender gap 10 µm** |
| Thickness L_SPAN_ | 105 µm | 83 µm |
| Specific surface area $a_{\mathrm{SPAN}}^{v}$ | 1$\cdot$10^5^ m^-1^ | 1$\cdot$10^5^ m^-1^ |
| Discretization units | 5 / 5 / 5 / 5 / 5 | 5 / 5 / 5 / 5 / 5 |
| Unit size | 0.1/0.95/18.9/0.95/0.1 µm | 0.083/0.75/15/0.75/0.083 µm |
|  |  |  |
|  | *Carbon / Binder (CB)-Phase* | |
| volume fraction $\varepsilon$_CB_ | 0.0411 | 0.052 |
|  |  |  |
|  | *SPAN-Phase* | |
| volume fraction $\varepsilon$_SPAN_ | 0.373 | 0.47 |
|  |  |  |
|  | *Li_2_S-Phase* | |
| volume fraction $\varepsilon_{Li_{2}S}$ | 1$\cdot$10^-4^ | 1$\cdot$10^-4^ |
| Li_2_S density $\rho_{Li_{2}S}$ | 1659 kg/m^3^ | 1659 kg/m^3^ |
| Surface exponent $\xi$ | 1.5 | 1.5 |
| Porosity $\varepsilon$_cat_ | 0.586 | 0.478 |
|  |  |  |
|  | **Separator** | |
| Thickness L_sep_ | 25 µm | |
| Discretization units | 5 | |
| Unit size | 5 µm | |
| volume fraction $\varepsilon$_sep_ | 0.45 | |
| Porosity | 0.55 | |
|  |  |  |
|  | **Li metal** | |
| volume fraction $\varepsilon$_an_ | 1 | |

**Table S 5.** Transport parameters of electrolyte and electrode materials. Transport parameters of the electrodes are standard parameters and deviations from standard values are indicated in the manuscript.

| Parameter | Value | Meaning | Ref. |
| --- | --- | --- | --- |
|  |  |  |  |
| **Electrolyte** | | | |
| $D_{\mathrm{LiA}}$ | 3.12$\cdot$10^-10^ m^2^/s | Li^+^, A^-^ diffusion coefficient | [7] |
| $D_{S^{2-}}$ | 6.1$\cdot$10^-11^ m^2^/s | S^2-^ diffusion coefficient | * |
| $t_{Li^{+}}$ | 0.1625 | Li^+^ transference number | [8] |
| $\kappa_{\mathrm{LiP}F_{6}}$ | 0.988 S/m | Electrolyte conductivity | [7] |
| $\left( 1+\frac{\partial\ln f_{e}}{\partial\ln c_{e}} \right)$ | 1.6 | Thermodynamic factor | [8] |
| $c_{Li^{+}}^{0}$ | 1000.02 mol/m^3^ | Li^+^ initial concentration | * |
| $c_{PF_{6}^{-}}^{0}$ | 1000 mol/m^3^ | A^-^ initial concentration | * |
| $c_{S^{2-}}^{0}$ | 1$\cdot$10^-5^ mol/m^3^ | S^2-^ initial concentration | * |
|  |  |  |  |
| **Solid** | | | |
| $\kappa_{\mathrm{SPAN}}^{\hom}$ | 2.5$\cdot$10^-2^ S/m | Conductivity homogeneous electrode | * |
| $\kappa_{\mathrm{SPAN}}^{\mathrm{het}}$ | 1$\cdot$10^-4^ S/m | Conductivity in heterogeneity |  |
| $\beta_{\mathrm{cat}}^{\hom}$ | 2.3 | Bruggeman in homogeneous electrode | * |
| $\beta_{\mathrm{cat}}^{\hom}$ | 10 | Bruggeman in heterogeneity |  |
| $\beta$_sep_ | 2.52 | Separator Bruggeman coefficient | [9] |
| $c_{\mathrm{DL}}$ | 0.1 F/m^2^ | Double layer capacity | [9] |

**Table S 6.** Standard parameters of kinetic model published by Simanjuntak et al.^[6]^ Reference and initial concentrations for discharge simulations of calendered electrodes with 10 µm calender gap are adjusted considering changes in electrode geometry as indicated in Table S 2.

| Parameter | Value | Meaning | Ref. |
| --- | --- | --- | --- |
|  |  |  |  |
| **S_4_ + 2e^-^+ 2 Li^+^ ⇌ S_3_Li + S_1_Li** | | | |
| $k_{f,S_{4}}^{0}$ | 1 mol/m^2^s | Frequency factor of reaction | [6] |
| $\alpha_{S_{4}}$ | 0.5 | Symmetry factor of transition state | [6] |
| $U_{S_{4}}^{eq,0}$ | 2.2 V | Parameters for the calculation of the SPAN OCP | ** |
| $b_{S_{4}}$ | -0.3 V |  | ** |
|  |  |  |  |
| **S_3_Li + 2e^-^ ⇌ S_2_Li +** $\mathbf{S}^{\mathbf{2-}}$ | | | |
| $k_{f,S_{3}\mathrm{Li}}^{0}$ | 1 mol/m^2^s | Frequency factor of reaction | ** |
| $\alpha_{S_{3}\mathrm{Li}}$ | 0.5 | Symmetry factor of transition state | ** |
| $U_{S_{3}\mathrm{Li}}^{eq,0}$ | 1.9 V | Parameters for the calculation of the SPAN OCP | ** |
| $b_{S_{3}\mathrm{Li}}$ | -0.28 V |  | ** |
|  |  |  |  |
| **S_2_Li + 2e^-^ ⇌ S_1_Li +** $\mathbf{S}^{\mathbf{2-}}$ | | | |
| $k_{f,S_{2}\mathrm{Li}}^{0}$ | 1$\cdot$10^-2^ mol/m^2^s | Frequency factor of reaction | ** |
| $\alpha_{S_{2}\mathrm{Li}}$ | 0.5 | Symmetry factor of transition state | ** |
| $U_{S_{3}\mathrm{Li}}^{eq,0}$ | 1.9 V | Parameters for the calculation of the SPAN OCP | ** |
| $b_{S_{2}\mathrm{Li}}$ | -0.62 V |  | ** |
|  |  |  |  |
| **Li^+^ + e^-^ ⇌ Li** | | | |
| $k_{f,\mathrm{Li}}^{0}$ | 3.94 mol/m^2^s | Frequency factor of reaction |  |
| $\alpha_{\mathrm{Li}}$ | 0.5 | Symmetry factor of transition state |  |
| $U_{\mathrm{Li}}^{eq,0}$ | -0.1775 | OCP at reference condition |  |
| **2Li^+^ + S^2-^ ⇌ Li_2_S** | | | |
| $K_{SP}^{Li_{2}S}$ | 10 | Solubility |  |
| $k_{f,\mathrm{Li}_{2}S}^{0}$ | $2\cdot$10^2^ mol/m^2^s | Growth factor |  |
| $c_{S_{4}}^{0}$ | 7.44$\cdot$10^3^ mol/m^3^ | PAN-S_4_ initial concentration (10 µm calender gap) |  |
| $c_{S_{3}\mathrm{Li}}^{0}$ | 1$\cdot$10^-5^ mol/m^3^ | PAN-S_3_Li initial concentration |  |
| $c_{S_{2}\mathrm{Li}}^{0}$ | 1$\cdot$10^-5^ mol/m^3^ | PAN-S_2_Li initial concentration |  |
| $c_{S_{1}\mathrm{Li}}^{0}$ | 1$\cdot$10^-5^ mol/m^3^ | PAN-SLi initial concentration |  |
| $c_{S_{4}}^{\mathrm{ref}}$ | 7.44$\cdot$10^3^ mol/m^3^ | PAN-S_4_ reference concentration (10 µm calender gap) |  |
| $c_{S_{3}\mathrm{Li}}^{\mathrm{ref}}$ | 7.44$\cdot$10^3^ mol/m^3^ | PAN-S_3_Li reference concentration (10 µm calender gap) |  |
| $c_{S_{2}\mathrm{Li}}^{\mathrm{ref}}$ | 7.44$\cdot$10^3^ mol/m^3^ | PAN-S_2_Li reference concentration (10 µm calender gap) |  |
| $c_{S_{1}\mathrm{Li}}^{\mathrm{ref}}$ | 1.487$\cdot$10^4^ mol/m^3^ | PAN-SLi reference concentration (10 µm calender gap) |  |





**Figure S 3.** Particle size distribution of commercially acquired SPAN and NMC 622 (as delivered), obtained via laser scattering measurements with a laser wave length of 650 nm. The measurement procedure is described in detail by Moschner et al. in [1].


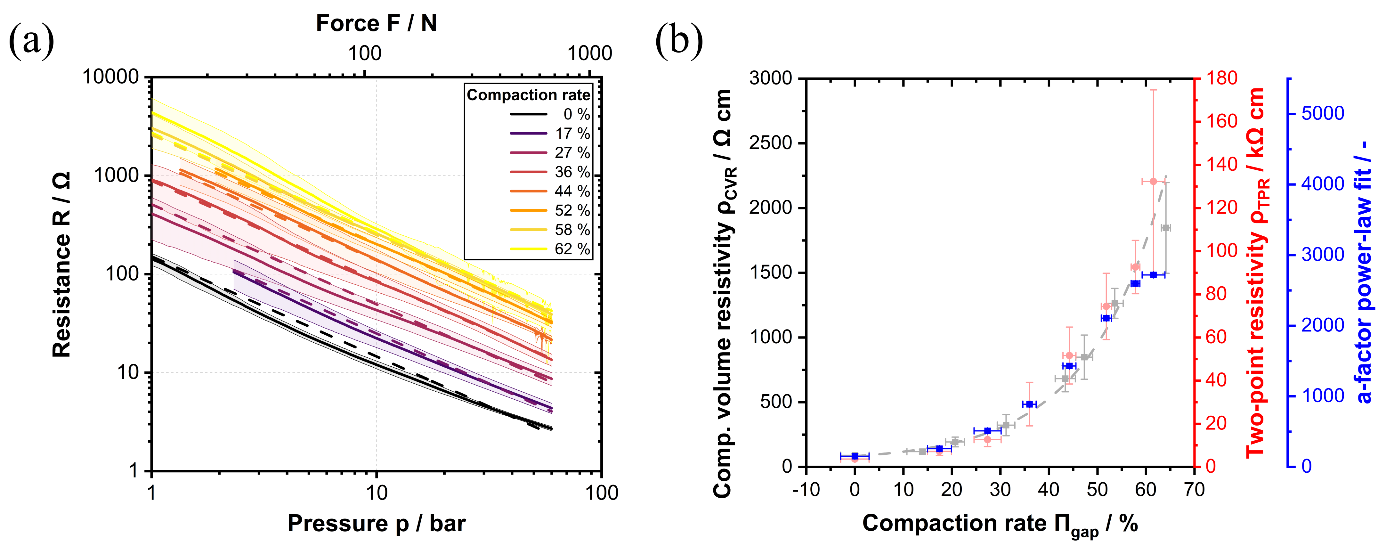


**Figure S 4.** Electronic properties of calendered SPAN electrodes: a) Pressure dependency fitted with power law y = a * x^b^, where b is fixed to -1.01594 (for fitting parameters, see **Table S 7**); b) Dependency of a-factor of power law fits from compaction rate of the SPAN electrode.

**Table S 7.** Detailed overview of the fitting parameters for the pressure dependent resistance of the different SPAN electrodes with power law y = a*x^b^

| Compaction rate Π_gap_ in % | Density  ρ_after_  in g cm^‑^³ | Free exponent b | | | Fixed exponent b | | |
| --- | --- | --- | --- | --- | --- | --- | --- |
|  |  | Factor  a | Exponent b | Adj. R² | Factor  a | Exponent b | Adj. R² |
| 0 | 0.707 | 137.4 | -1.055 | 0.995 | 151.1 | -1.016 | 0.902 |
| 17.45 | 0.793 | 187.2 | -0.922 | 0.998 | 258.7 |  | 0.976 |
| 27.36 | 0.802 | 352.3 | -0.904 | 0.999 | 511.2 |  | 0.955 |
| 35.99 | 0.833 | 905.9 | -1.022 | 0.998 | 886.8 |  | 0.998 |
| 44.23 | 0.868 | 1538.7 | -1.036 | 0.998 | 1430.1 |  | 0.998 |
| 51.83 | 0.905 | 2202.1 | -1.029 | 0.998 | 2107.3 |  | 0.998 |
| 57.83 | 0.919 | 2842.8 | -1.042 | 0.998 | 2596.9 |  | 0.997 |
| 61.55 | 0.948 | 3674.5 | -1.118 | 0.989 | 2719.1 |  | 0.967 |


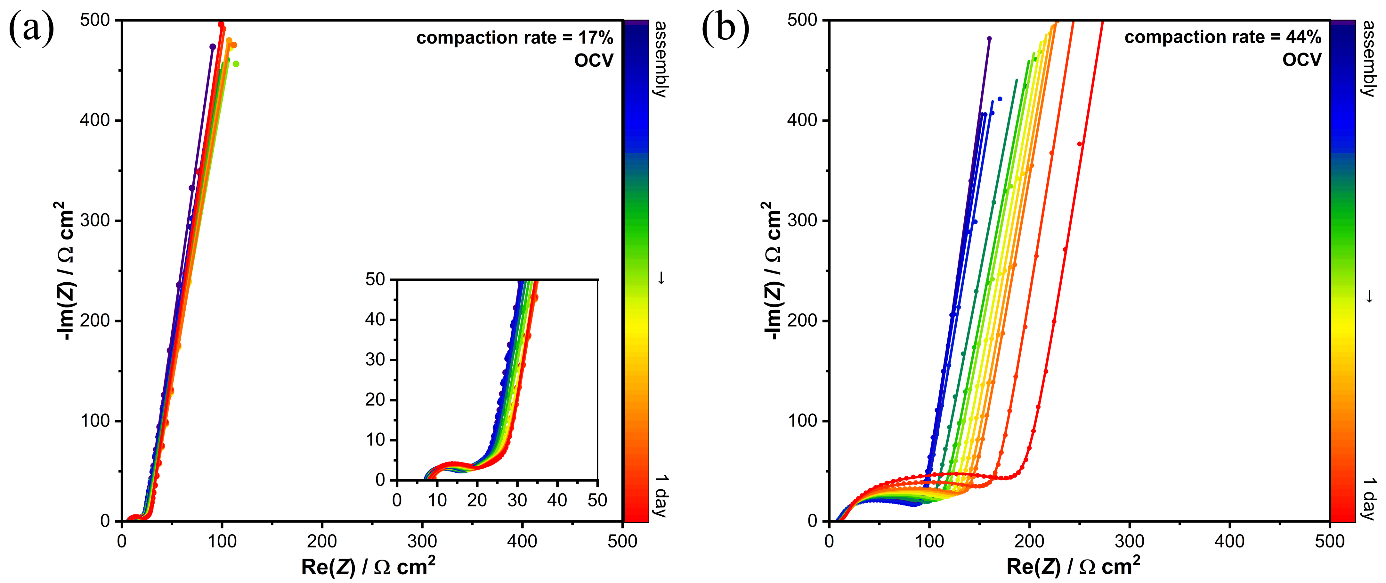


**Figure S 5.** OCV impedance spectra of a) 17 % and b) 44 % calendering grade


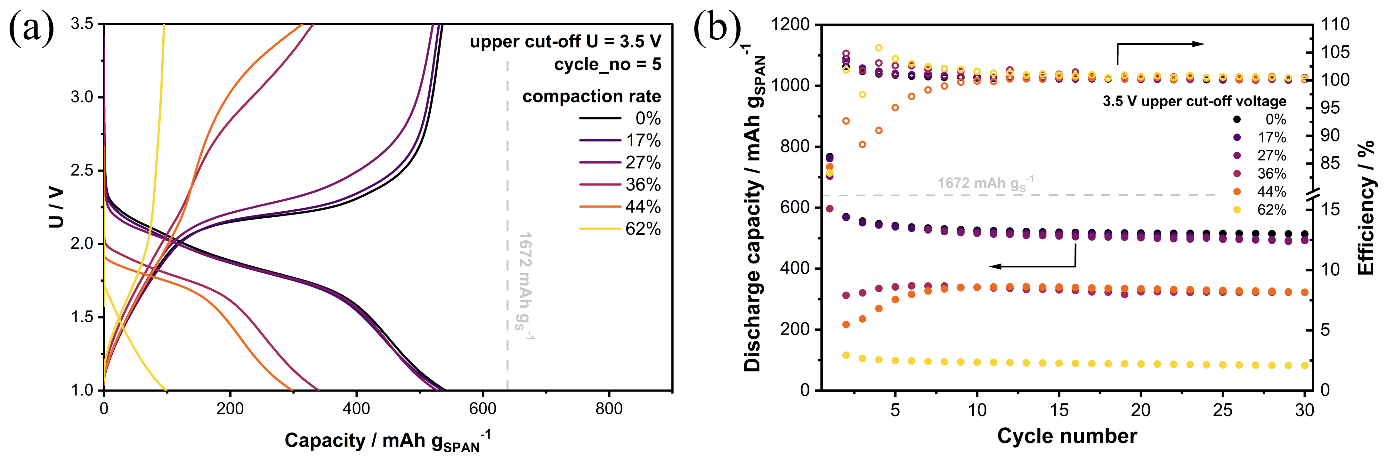


**Figure S 6**. a) Voltage profile comparison of further compression rates at an upper cut-off voltage of 3.5 V and b) corresponding capacity/efficiency values.





**Figure S 7.** Capacity and coulombic efficiency of different calendering grades cycled up to 3.0 V with C/10 and C/3


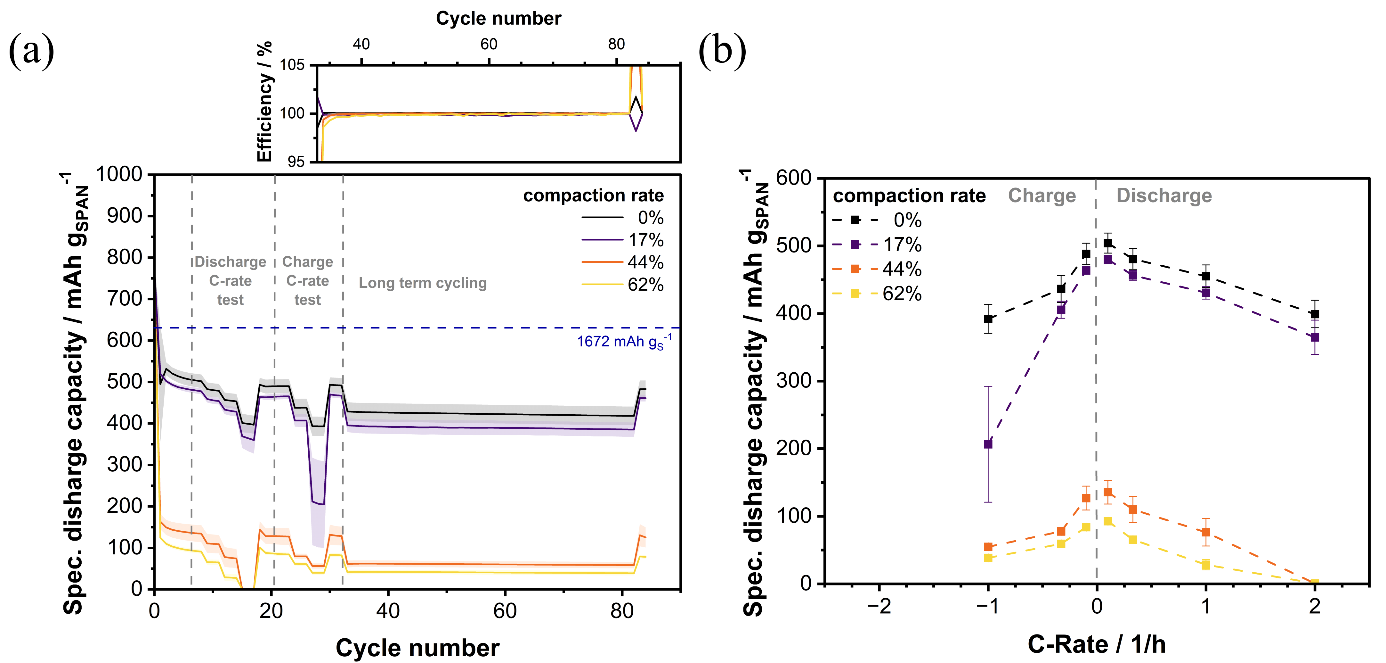


**Figure S 8.** Discharge and charge C-rate tests as well as long term cycling tests for various calendering grades: a) specific discharge capacity per cycle; b) evaluation specific discharge capacity depending on the C-rate





**Figure S 9.** Simulated impedance spectra for varying Bruggeman constants determining the ionic resistance in the porous electrodes.

1. **Assignment of high-, mid- and low-frequency inter-particle processes**

The assignment of the high-, mid- and low-frequency inter-particle processes is performed by differentiation of their relaxation times *τ*, which are mathematically linked via the effective capacitance and resistance:

$\tau=R\cdot C_{eff}=\frac{1}{f}$ ,

whereby the effective capacity *C*_eff_ is calculated using the exponential factor α:

$C_{\mathrm{eff}}=Q^{\frac{1}{\alpha}}{*R}^{\frac{\left( 1-\alpha\right)}{\alpha}}$ [10].

The calculated effective capacitance values, time constants and frequency ranges of the fitted processes as well as their assignment can be found in **Figure S 10** and **Table S 8**. The assignment is made as follows:

- Process 1: inter-particle effects with small relaxation time constant 🡪 Q/R_int1_ (grey)
- Process 2: inter-particle effects with medium relaxation time constants 🡪 Q/R_int2_ (purple)
- Process 3: inter-particle effects with high relaxation time constants 🡪 Q/R_int3_) (orange)





**Figure S 10.** Fitted time constants of the various RQ circuits for different calendering grades and process assignment

**Table S 8.** Fitted time constants, characteristic frequencies and effective capacitance values for the fitted processes

|  | **comp. rate = 62%** | | | **comp. rate = 44%** | | | **comp. rate = 17%** | | | **Uncalendered** | | |
| --- | --- | --- | --- | --- | --- | --- | --- | --- | --- | --- | --- | --- |
|  | f [Hz] | τ [s] | C_eff_ [F] | f [Hz] | τ [s] | C_eff_ [F] | f [Hz] | τ [s] | C_eff_ [F] | f [Hz] | τ [s] | C_eff_ [F] |
| **QR_int1_** |  |  |  |  |  |  | 6.2E3 ±7.5E4 | 1.2E-6 ±1.3E-5 | 9.9E-9 ±5.8E-7 | 9.9E4 ±4.7E3 | 1E-5 ±5E-7 | 5.8E-7 ±2.8E-8 |
| **QR_int2_** | 1.8E3 ±5.1E2 | 6.5E-4 ±3.3E-4 | 2.3E-6 ±5.1E-7 | 4.4E3 ±8.3E2 | 2.4E-4 ±5.4E-5 | 1.2E-6 ±6.4E-8 |  |  |  |  |  |  |
| **QR_int3_** | 8.9E1 ±3.3E1 | 1.4E-2 ±9E-3 | 1.7E-5 ±3.5E-6 | 3.1E2 ±4.6E1 | 3.3E-3 ±5.9E-4 | 4.5E-5 ±1E-5 |  |  |  |  |  |  |

References

[1] R. Moschner, H. Cavers, P. Michalowski, A. Kwade*, Batter. Supercaps* **2024**.

[2] A. Diener, S. Ivanov, W. Haselrieder, A. Kwade*, Energy Technol.* **2022**, 10.

[3] L. Froboese, P. Titscher, B. Westphal, W. Haselrieder, A. Kwade*, Mater. Charact.* **2017**, 133, 102.

[4] W. Haselrieder, B. Westphal, H. Bockholt, A. Diener, S. Höft, A. Kwade*, Int. J. Adhes. Adhes.* **2015**, 60, 1.

[5] B.G. Westphal, N. Mainusch, C. Meyer, W. Haselrieder, M. Indrikova, P. Titscher, H. Bockholt, W. Viöl, A. Kwade*, J. Energy Storage* **2017**, 11, 76.

[6] E.K. Simanjuntak, T. Danner, P. Wang, M.R. Buchmeiser, A. Latz*, Electrochim. Acta* **2024**, 497, 144571.

[7] G. Horwitz, M. Factorovich, J. Rodriguez, D. Laria, H.R. Corti*, ACS omega* **2018**, 3, 11205.

[8] H. Lundgren, M. Behm, G. Lindbergh*, J. Electrochem. Soc.* **2015**, 162, A413-A420.

[9] J. Landesfeind, J. Hattendorff, A. Ehrl, W.A. Wall, H.A. Gasteiger*, J. Electrochem. Soc.* **2016**, 163, A1373-A1387.

[10] B. Hirschorn, M.E. Orazem, B. Tribollet, V. Vivier, I. Frateur, M. Musiani*, Electrochim. Acta* **2010**, 55, 6218.
